# Supplementary material for: Experimental observation of localized interfacial phonon modes
Source: Nat Commun. 2021 Nov 25;12:6901. doi: 10.1038/s41467-021-27250-3 (PMC8617064; doi:10.1038/s41467-021-27250-3)
Supplement: Supplementary file 1 — Supplementary Information [file 41467_2021_27250_MOESM1_ESM.pdf]

Supplementary Information

Experimental observation of localized interfacial phonon modes

Zhe Cheng[1,10,11], Ruiyang Li[2,11], Xingxu Yan[3,4,11], Glenn Jernigan[5], Jingjing Shi[1], Michael E. Liao[6], Nicholas J. Hines[1], Chaitanya A. Gadre[7], Juan Carlos Idrobo[8], Eungkyu Lee[9], Karl D. Hobart[5], Mark S. Goorsky[6], Xiaoqing Pan[3,4,7,*], Tengfei Luo[2,*], Samuel Graham[1,*]

[1] George W. Woodruff School of Mechanical Engineering, Georgia Institute of Technology, Atlanta, GA 30332, United States

[2] Department of Aerospace and Mechanical Engineering, University of Notre Dame, Notre Dame, IN 46556, United States

[3] Department of Materials Science and Engineering, University of California, Irvine, CA 92697, United States

[4] Irvine Materials Research Institute, University of California, Irvine, CA 92697, USA.

[5] U.S. Naval Research Laboratory, 4555 Overlook Avenue SW, Washington, DC 20375, United States

[6] Materials Science and Engineering, University of California, Los Angeles, Los Angeles, CA 90095, United States

[7] Department of Physics and Astronomy, University of California, Irvine, CA 92617, United States.

[8] Center for Nanophase Materials Sciences, Oak Ridge National Laboratory, Oak Ridge, TN 37831, United States

[9] Department of Electronic Engineering, Kyung Hee University, Yongin-si, Gyeonggi-do 17104, South Korea

[10] Present address: Department of Materials Science and Engineering, University of Illinois at Urbana-Champaign, Urbana, IL 61801, United States.

[11] These authors contributed equally: Zhe Cheng, Ruiyang Li, Xingxu Yan.

[*]Corresponding authors: sgraham@gatech.edu; tluo@nd.edu; xiaoqinp@uci.edu

Supplementary Note 1: Materials growth

RCA cleaning procedure is a standard set of wafer cleaning steps which need to be performed before high-temperature processing steps of Si wafers. The standard recipe includes Standard Clean-1 for organic and particle clean, Standard Clean-2 for ionic clean, and final step for rinsing and drying. The growth temperature profile of Sample 1 is shown in Supplementary Fig. 1a. Sample 1 was used for EELS and Raman measurements. The growth temperature profile of Sample 2 is shown in Supplementary Fig. 1b. The growth of Sample 1 was designed to create a Ge film with the fewest amount of threading dislocations while obtaining a relaxed Ge lattice parameter. The Ge film has fully relaxed to a 5.68 Å lattice parameter for Ge. That is why the Raman spectrum of the Ge film lines up with that of the Ge wafer. The growth of Sample 2 without cap was optimized for TDTR measurements (Supplementary Fig. 12), where the Ge film was grown at a constant temperature leading to a uniform film but expect a higher number of threading dislocations.

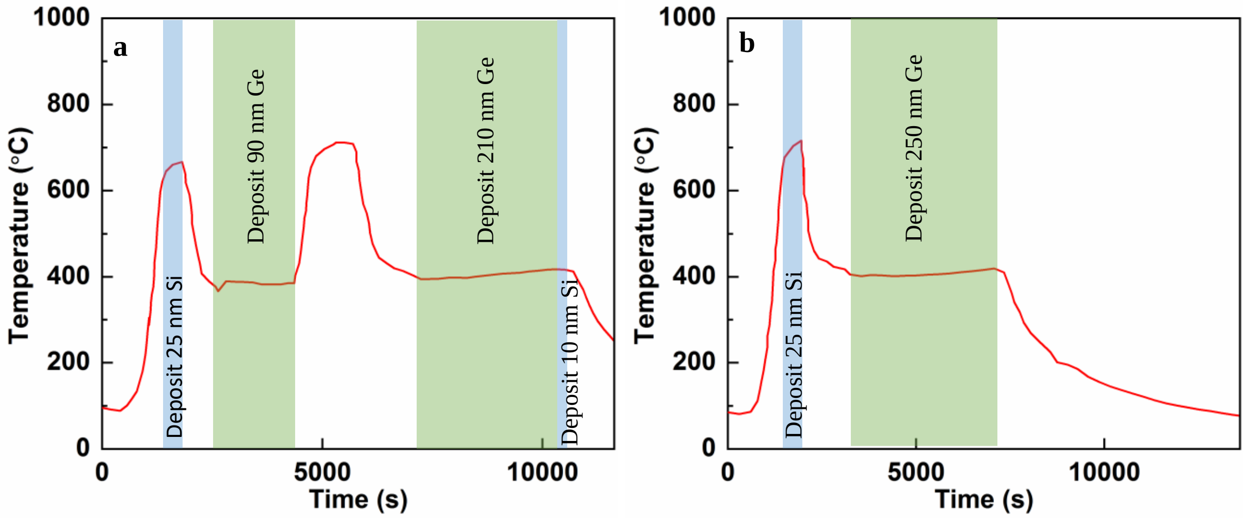

Supplementary Fig. 1 Profiles of growth temperatures. a Growth temperature of Sample 1 (Si-Ge-Si). **b** Growth temperature of Sample 2 (Ge-Si).

Supplementary Note 2: Thermal and XRD characterizations

The schematic diagram of the sample structure for TDTR measurements is shown in Supplementary Fig. 2a. Validation samples (e.g., single crystal Si) were measured to check the TDTR system. The Al thermal conductivity is obtained by measuring its electrical conductivity and applying the Wiedemann-Franz law. The Al and Ge thicknesses were determined by picosecond acoustic technique. The heat capacity of Ge, the heat capacity and thermal conductivity of the Si substrate are from literature.[1-3] The slight boron doping with a concentration of 5×10^{14}-1×10^{15} atoms/cm^3 does not affect the thermal conductivity of the Si substrate at room temperature and above,[4] although it may affect the thermal conductivity of Si wafers below 40 K.[4] The error bars were calculated based on a Monte Carlo method.[5] All the related uncertainty sources are considered in the calculation. The error of laser spot sizes is ±0.5 μm. All the relative errors of heat capacity are ±2%. The relative error of Al thermal conductivity is ±10% and the error of thicknesses determined by the picosecond acoustic technique is ±3 nm. The measured Ge thermal conductivity is close to the literature value of Ge films.[6] Supplementary Fig. 2b show the TDTR sensitivity of Ge thermal conductivity and Si-Ge TBC at room temperature with the TDTR system at GT.

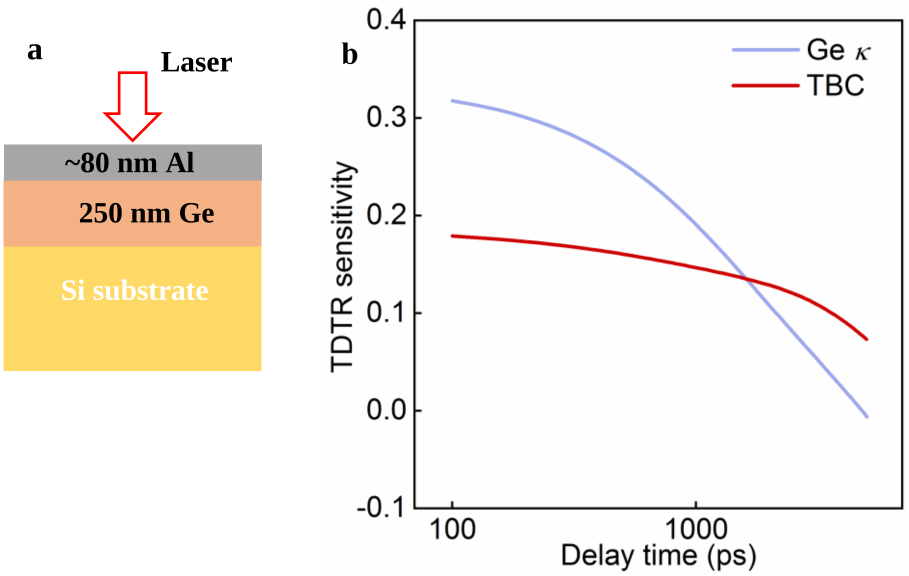

Supplementary Fig. 2. Time-domain thermoreflectance (TDTR) measurements on Sample 2. **a** Schematic diagram of TDTR measurements. **b** The TDTR sensitivities of the Si-Ge TBC and the thermal conductivity of the Ge layer (Ge *k*).

Supplementary Fig. 3 shows the high-resolution X-ray diffraction (XRD) results of Sample 2: triple-axis ω:2θ line scan through the (004) symmetric planes and triple-axis asymmetric 224 reciprocal space map (RSM) measured in the glancing exit geometry. In the symmetric line scan shown in Supplementary Fig. 3a, the green dotted line shows the fully relaxed (004) Ge position relative to (004) Si. The full width at half maximum (FWHM) of the Si peak is 15” ±2” while the FWHM of the Ge peak is 200” ±25”. The relaxation values from the ω:2θ scan for the 250-nm Ge are 100.0% ± 0.7%. This is in agreement with the (224) asymmetric RSM shown in Supplementary Fig. 3b, which shows a relaxation of 99.8% ± 0.3%. The major axis of the 224 Ge reciprocal lattice point is tilted ~35° from the horizontal. This matches the interplanar angle of 35.2° between (004) and (224), which suggests that the peak broadening is dominated by lattice mosaicity in the Ge layer.

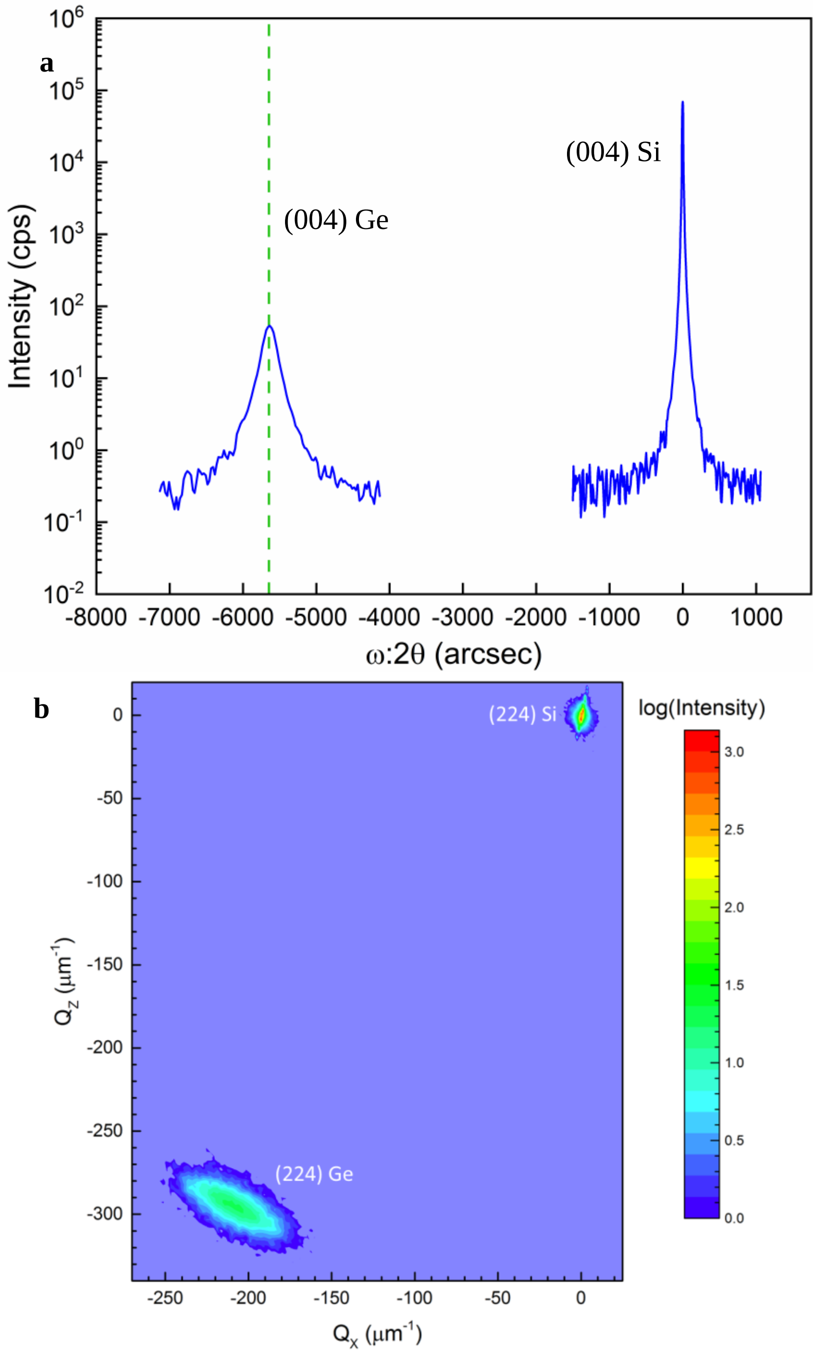

Supplementary Fig. 3. The X-ray diffraction (XRD) results of Sample 2. a Triple-axis symmetric (004) ω:2θ scan where the green dotted line shows the fully relaxed (004) Ge position

relative to (004) Si. **b** Triple-axis asymmetric 224 reciprocal space map performed in the glancing exit geometry.

Supplementary Note 3: EELS data

To further confirm that the intensity peak shown in Fig. 2d of the main text is contributed by the interfacial mode, a linear regression fitting of the EELS data at the interface is performed, as shown in Supplementary Fig. 4. The overlapping of the Si signal and Ge signal cannot produce an intensity peak near 12.0 THz. The intensity peaks near 12.0 THz in Fig. 2d of main text are contributed by the interfacial mode.

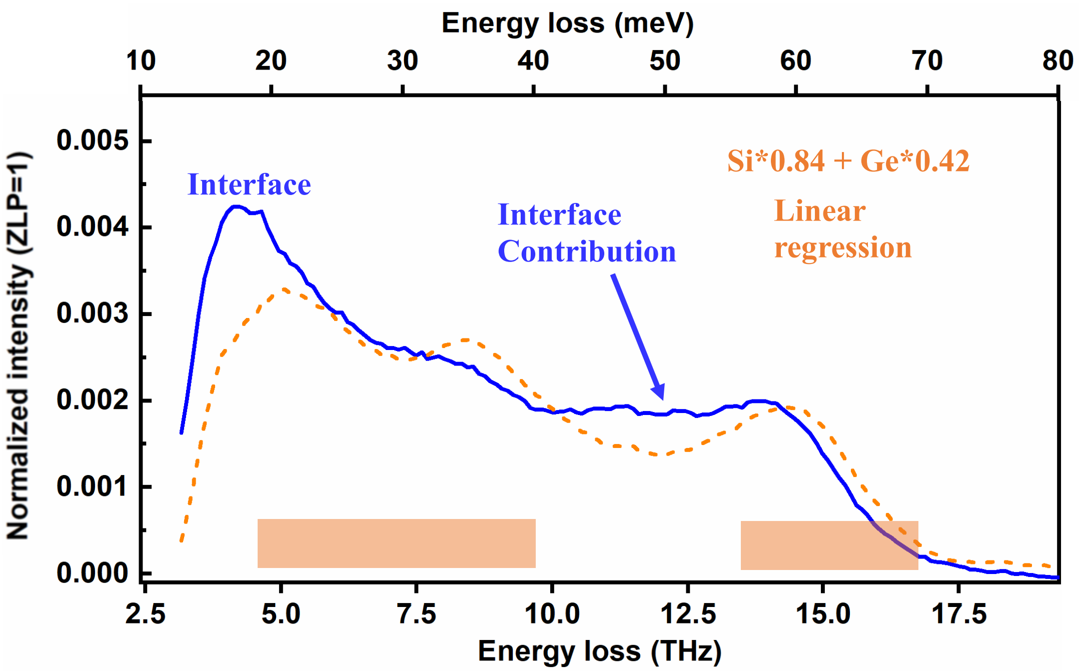

Supplementary Fig. 4. Linear regression fitting of the EELS signal at the interface. The overlapping of the Si signal and Ge signal cannot produce an intensity peak near 12.0 THz. The intensity peaks near 12.0 THz in Fig. 2d of the main text are contributed by the interfacial modes.

Supplementary Note 4: Non-equilibrium Landauer approach

TBC was calculated by both non-equilibrium and conventional Landauer approaches for comparison. Here, diffuse mismatch model (DMM) was used to calculate the transmission coefficient for DMM-based TBC.[7,8] The phonon dispersion relations of Si and Ge used to obtain transmissions were calculated by density functional theory (DFT). Radiation limit (RL) was also calculated with both non-equilibrium and conventional Landauer approaches.[9,10] RL assumes 100% transmission in the elastic channel. The detailed description about how to use the non-equilibrium Landauer approach to calculate DMM-based TBC and RL TBC can be found in literature.[10] Supplementary Fig. 5 shows the comparison of the RL TBC and DMM-based TBC with/without considering the non-equilibrium effect. By considering the non-equilibrium effect, the corrected RL and DMM-based TBC are higher than their conventional counterparts.

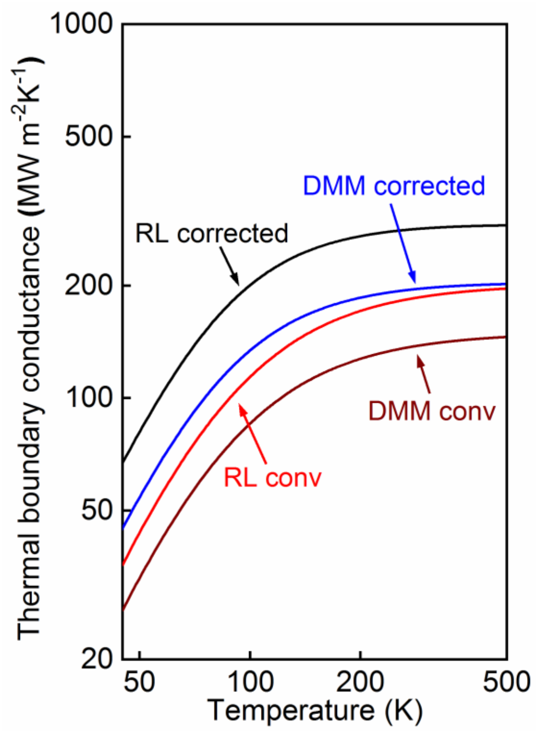

Supplementary Fig. 5. The comparison of the radiation limit (RL) TBC and DMM-based TBC with/without considering the non-equilibrium effect. By considering the non-equilibrium effect, the corrected RL and DMM-based TBC are higher than their conventional counterparts.

Supplementary Note 5: Neural network potential construction

Non-equilibrium molecular dynamics (NEMD) simulations of the Si-Ge interfaces were performed to understand the thermal transport across the Si-Ge interfaces by using a neural network-based interatomic potential. Compared with empirical potentials (e.g. Tersoff[11] and Stillinger-Weber[12] potentials), neural network potentials (NNP) can accurately reproduce the *ab-initio* energies and forces with a larger interaction cutoff. Such potentials have been used to study thermal properties over a wide range of materials, such as Si in various phases, MoS_2, and Ga_2O_3.[13-16] Supplementary Fig. 6a shows the comparison of *ab-initio* (AI) MD energies and forces and NNP predicted quantities of structures in the testing dataset, indicating that the NNP accurately reproduces the ab-initio energies and forces.

Supplementary Note 6: Non-equilibrium molecular dynamics simulations

NEMD simulations were performed using the LAMMPS package[17] with this NNP for the Si-Ge interfaces, as shown in Supplementary Fig. 6b. A typical non-equilibrium temperature profile observed in this case is shown in Supplementary Fig. 6c.

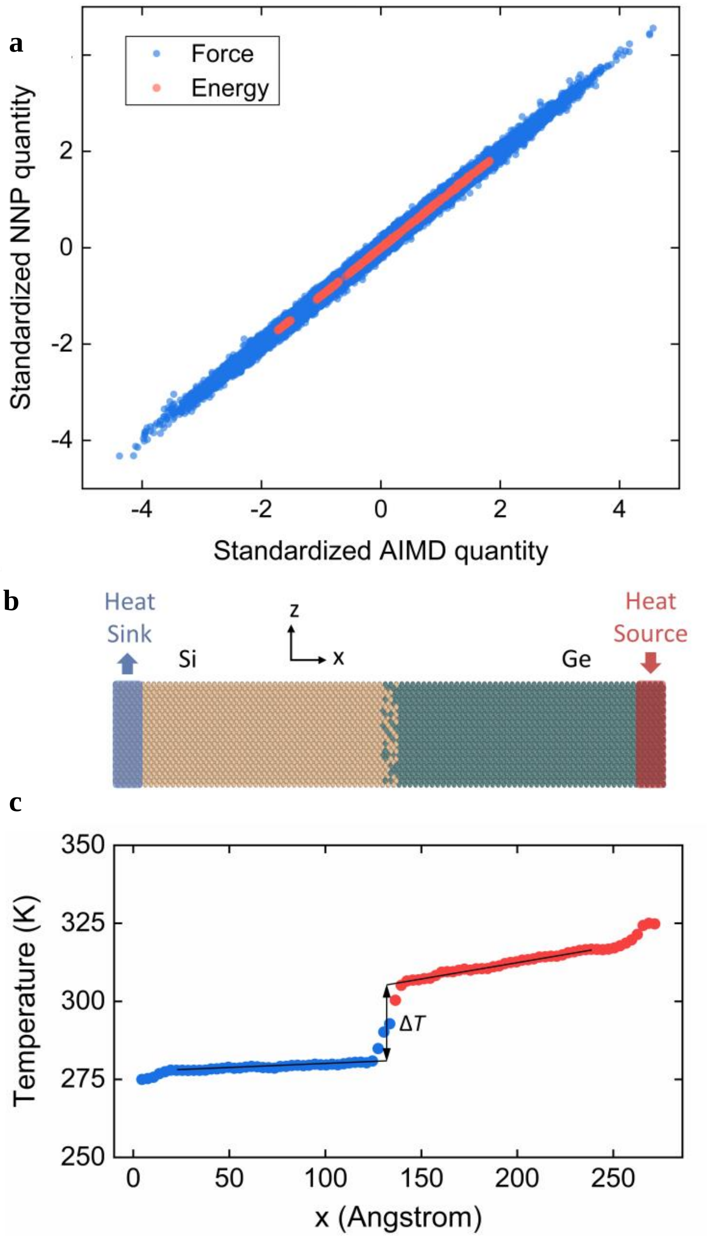

Supplementary Fig. 6. Non-equilibrium molecular dynamics (NEMD) simulations of the Si-Ge interfaces. a Standardized potential energies and atomic forces from MD simulations using NNP and ab-initio calculations. Energies and forces are standardized by subtracting the average (μ) from them and then divided by the standard deviation (σ). Both quantities are plotted within $\pm 5\sigma$. **b** Structure of an example of the Si-Ge interface with a 0.7 nm-thick mixed region at the

interface. The left and right ends are fixed during simulations. **c** The calculated steady-state temperature profile at 300 K along the direction perpendicular to the Si-Ge interfaces.

It has been reported that Si atoms encapsulated by Ge atomic cages can lead to interfacial modes.[18] For comparison, an ideally perfect Si-Ge interface is also calculated by MD with NNP. The interfacial modes also show up at the interface. As shown in Supplementary Fig. 7, we observed obvious peaks in the phonon density of states around 12 THz for the sharp Si-Ge interface. The Si atoms for the sharp interface have Si-Ge bonds but are not encapsulated by surrounding Ge atoms. The origin of the interfacial modes is thus believed to be from the Si-Ge bonds that do not exist in bulk Si or bulk Ge. Therefore, the vibrations at ~ 12 THz stem from the Si-Ge bonds where the two atoms tend to vibrate differently due to the mass difference compared to the atoms in bulk Si or bulk Ge. When the Si-Ge mixture is introduced at the interface, there are more Si-Ge bonds. Si encapsulated by Ge cages is one of such cases. In these cases, interfacial modes exist on atoms in the whole mixed region, as depicted by the eigenvectors of an interfacial mode in Fig. 5d for the interface with atomic mixing.

Additionally, the interfacial modes get occupied in a similar way as optical modes.[19] At a given temperature, the phonon distribution follows Bose-Einstein distribution which only depends on the phonon frequency. We note that the interfacial modes, which are mainly localized around the interface and have a high frequency of ~12 THz, are originated from the Si-Ge bonds unique to the interfacial region. The vibration of this bond is an intra-unit cell phenomenon, so it is similar to the origin of optical phonons. It is found that the eigenvectors are mostly localized in the mixed region, and only a few of them spatially extend to the first or second layer next to the intermixing

layers, which is consistent with the PDOS shown in Fig 3. Therefore, energy transport velocity is not well-defined for these interfacial modes which are localized, and the energy is transferred across the interface mainly by the coupling between interfacial modes and other propagating modes. This has been theorized in the literature.[20,21]

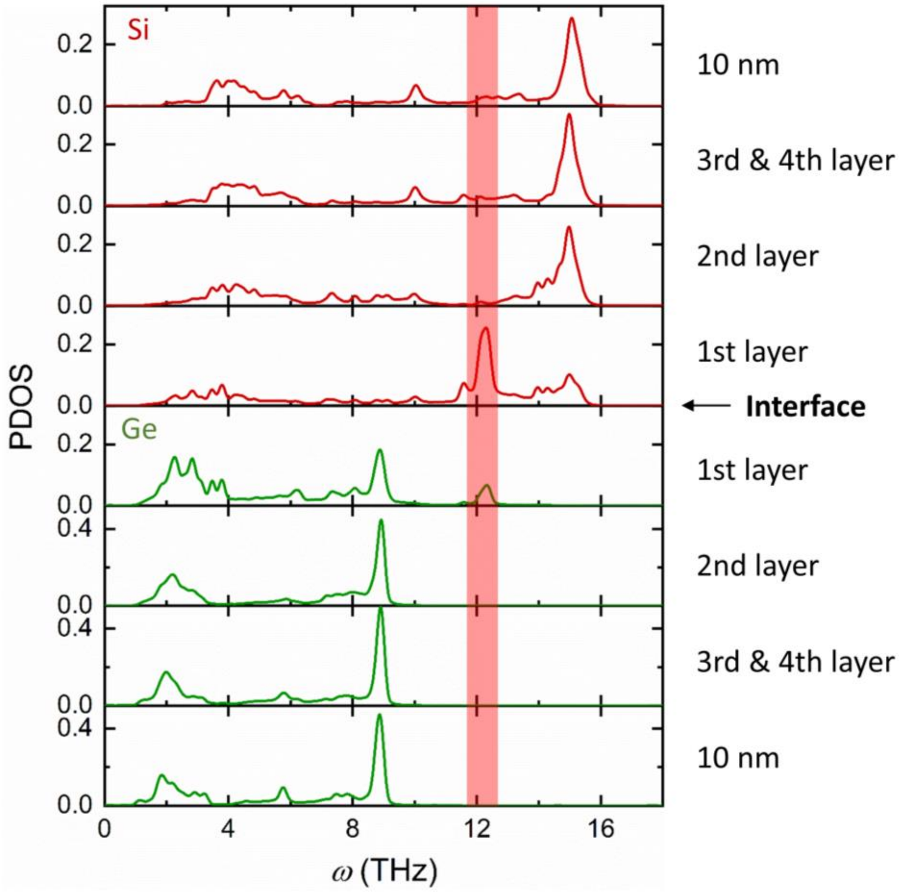

Supplementary Fig. 7. Phonon density of states (PDOS) for an ideally sharp Si-Ge interface at different locations throughout the domain along the direction perpendicular to the Si-Ge interface for comparison. Each panel is the PDOS of the atoms at a certain location with the distance from the interface labeled on the right of each panel.

Supplementary Note 7: Temperature dependent TDTR measurements

Supplementary Fig. 8 shows the sensitivities of Si-Ge TBC and Ge thermal conductivity as a function of delay time and temperature. The sensitivity of Si-Ge TBC decreases as temperature increases while the sensitivity of Ge thermal conductivity increases slightly as temperature increases. Typically, the TDTR measurements are reliable once the sensitivity of the unknown parameter is larger than 0.1. The TBC and Ge thermal conductivity values measured by the two TDTR systems are consistent. Please note that the error bar of the measured Si-Ge TBC calculated by the Monte Carlo method in this work considers all the possible error sources, including the effect from the Ge thermal conductivity in the data fitting. The error bars are similar for both TDTR systems at room temperature. For high temperature measurements, the sensitivity of TBC decreases. But the error bars from other parameters become smaller, such as the heat capacity of each layer and the Si thermal conductivity, because heat capacity values are less sensitive to temperature at high temperatures and Si thermal conductivity is less sensitive to defect scatterings due to increased anharmonic scattering. We thus obtained similar error bars for the reported TBC values at high temperatures. As shown in Supplementary Fig. 8c, the thermal conductivity of bulk Ge is also included for comparison.[22] The thermal conductivities of both the Ge thin film and bulk Ge decrease as temperature increases due to increased phonon-phonon scatterings. The reduced thermal conductivity of the Ge thin film compared to thermal conductivity of bulk Ge is due to size effect.

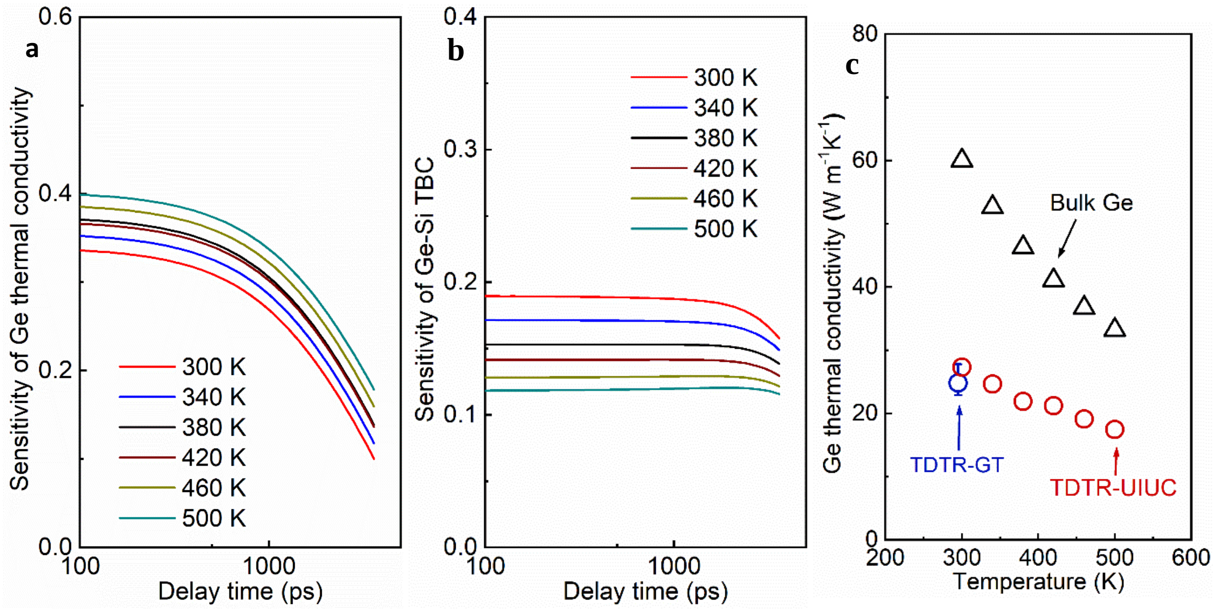

Supplementary Fig. 8 Temperature dependent thermal measurements. a Sensitivity of Ge thermal conductivity as a function of delay time and temperature. **b** Sensitivity of Si-Ge TBC as a function of delay time and temperature. **c** The measured thermal conductivity of the 250-nm Ge thin film (TDTR-GT and TDTR-UIUC) and bulk Ge at different temperatures. The reduction in thermal conductivity of the Ge thin film compared to the bulk thermal conductivity is due to size effect.[22] The error bar is calculated by a Monte Carlo method which considers all the possible error sources.

Supplementary Note 8: STEM measurements of interfaces

We did Raman and EELS measurements on one sample and TDTR measurements on the other sample because the Raman measurements need optical access to the interface, so the top Si layer needs to be very thin. On the other hand, TDTR measurements need a thick Ge layer. These factors prevent us from performing all the measurements on a single sample. However, the epitaxial Si-Ge interfaces are expected to be similar. To show that the two interfaces are similar, we characterized the Si-Ge interface of the TDTR sample using atomic resolution STEM, as shown

in Supplementary Fig. 9. The Si-Ge interface of the TDTR sample has a similar epitaxial growth structure as the interface used for Raman and EELS measurements.

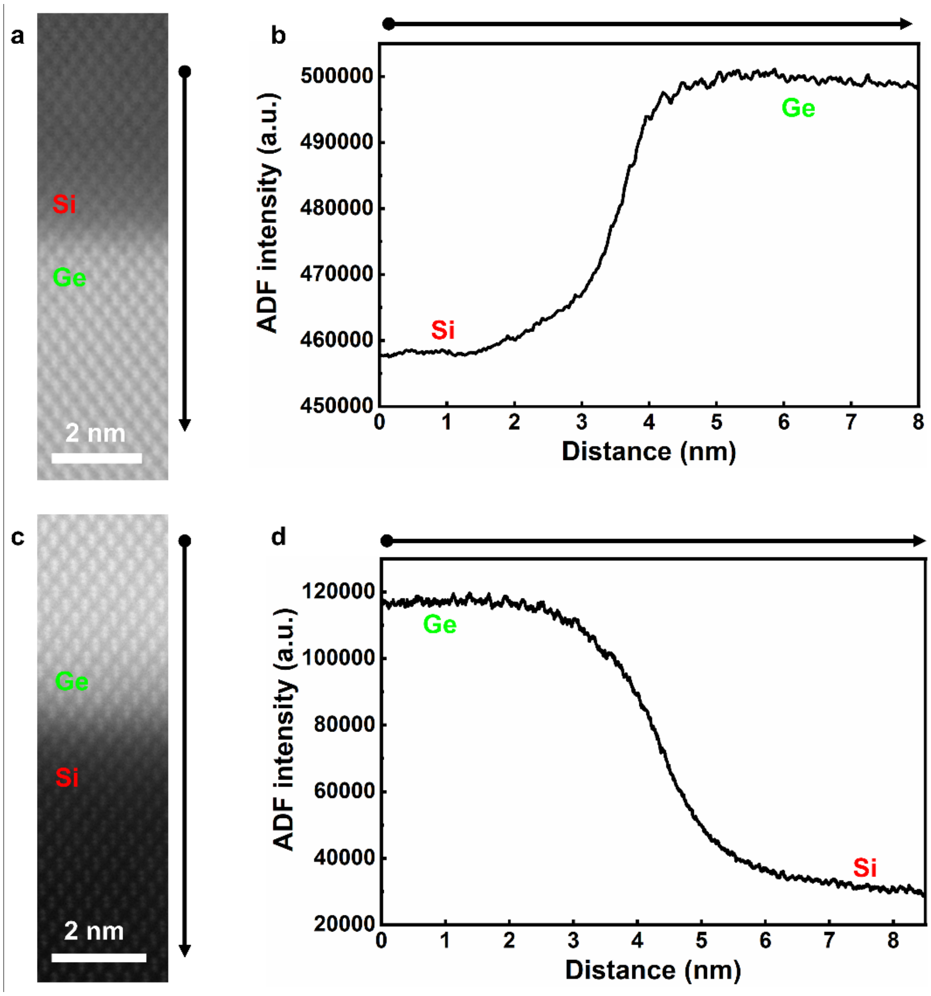

Supplementary Fig. 9 Interfacial structures of the two samples. a Atomic resolution STEM image of the Si-Ge interface for the Raman and EELS measurements. **b** The annular dark-field (ADF) intensity to show the atomic mixing at the interface. **c** Atomic resolution STEM image of the Ge-Si interface of the sample for TDTR measurements. **d** ADF intensity across the interface.

Previous theoretical calculations only consider the ideal Si-Ge interfaces, but realistic interfaces cannot be perfect interfaces. The intermixing during epitaxial growth of Si and Ge is typically un-

avoidable due to the high temperature and strain.[23,24] Decreasing growth temperature can reduce the atomic diffusion responsible for interfacial intermixing.[25] That is why we grew the samples at a relatively low temperature (400 °C), but it is still un-avoidable to have limited intermixing. To examine the intermixing between Si and Ge, we conducted atomic-resolution STEM imaging and energy-dispersive X-ray spectroscopy (EDS) mapping. Supplementary Fig. 9 shows the atomic-resolution high-angle annular dark-field scanning transmission electron microscopy (HAADF-STEM) image at the Si-Ge interface and intensity line profile across the interface. Since the HAADF signal monotonically increases with the atomic number (Z contrast) of the observed materials, we can use HAADF intensity to distinguish Si and Ge. Thus, the darker and brighter regions in Supplementary Fig. 9a are attributed to Si and Ge, respectively. Supplementary Figs. 10a-c show STEM-EDS results acquired at the interface. Using the line profile of both Si and Ge proportions in Supplementary Fig. 10d, we observed interfacial intermixing as well.

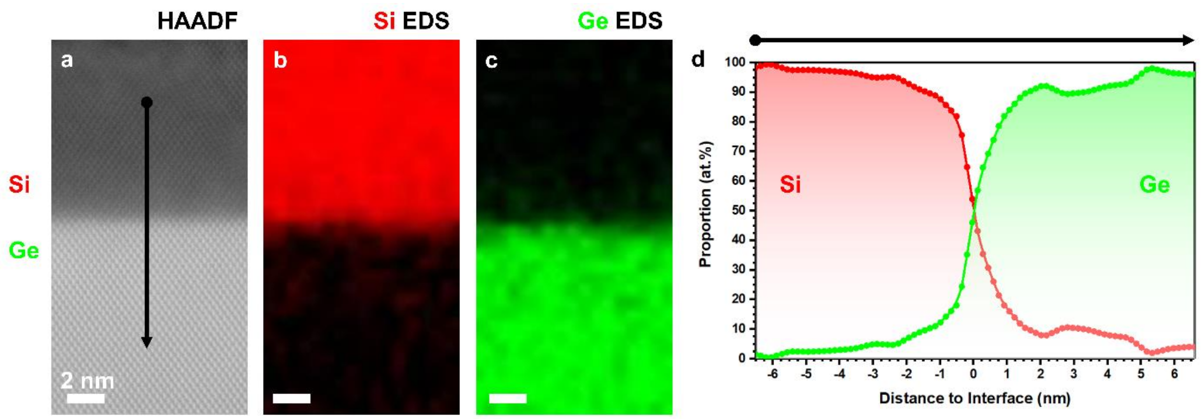

Supplementary Fig. 10 Energy dispersive X-ray spectroscopy (EDS) mapping of the Si-Ge interface of the Raman and EELS sample. a HAADF-STEM image acquired at the Si-Ge interface. **b, c** EDS elemental mapping of Si (b, red) and Ge (c, green) from the same region in a. Scale bars in (a-c) are all 2 nm. **d** Line profiles of atomic percentage of Si and Ge along the arrow in **a**.

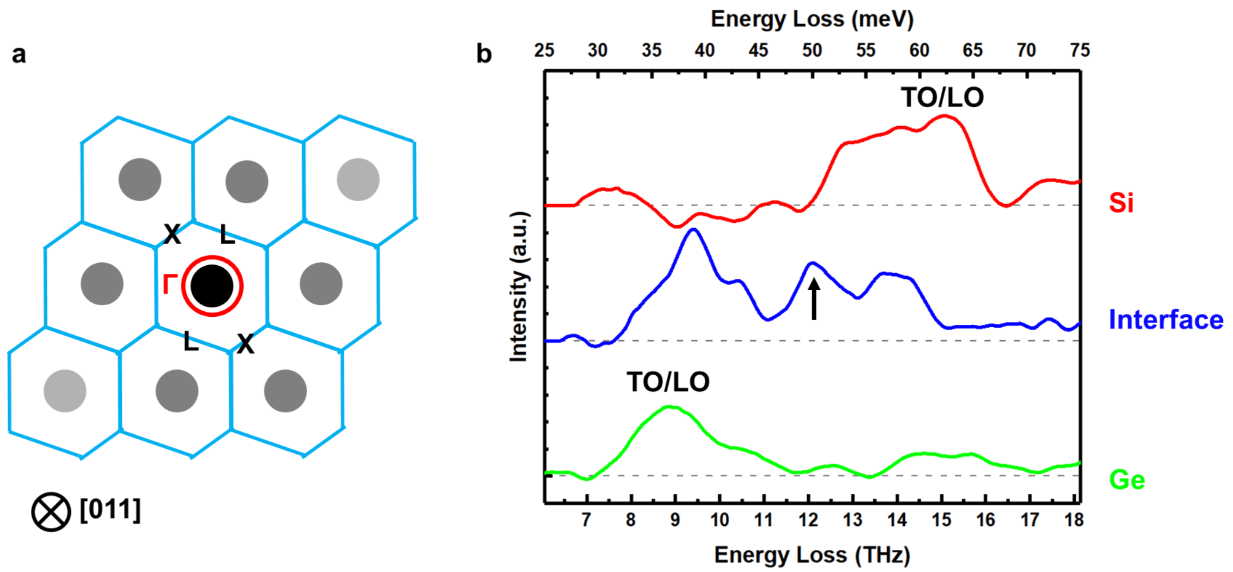

Supplementary Fig. 11 Momentum resolved vibrational spectra in the Si-Ge heterointerface with a convergence semi-angle of 3 mrad. a Reciprocal space diagram including the convergent beam diffraction pattern (CBED) of Si/Ge along [011] direction and the BZs (cyan contours). The center black disk and surrounding gray ones are the transmitted beam and diffracted beams with a radium of 3 mrad. The red circle represents the EELS entrance aperture, which positions at the BZ center to collect the corresponding phonon signals using this angle-resolved condition. The locations of Γ, X, and L points are indicated. **b** Local angle-resolved vibrational spectra of Si, Ge and interface. Three dashed horizontal lines are the zero baseline.

Supplementary Note 9: Momentum-resolved EELS measurements

Using a combination of a small convergence semi-angle (3 mrad) and a small EELS collection angle, we can obtain the angle-resolved vibrational signal with high momentum resolution (0.5 $Å^{-1}$) and appropriate spatial resolution (2.6 nm).[26] Supplementary Fig. 11b depicts local angle-resolved vibrational spectra acquired at the bulk Si, bulk Ge and interface. Si and Ge spectra contain one major peak at 15.1 THz (62.4 meV) and 8.9 THz (36.8 meV), respectively, which are

consistent with the optical phonon modes at the BZ center of Si and Ge.[27,28] There are additional phonon modes at ~12.0 THz in the interfacial vibrational spectra and shifted bulk optical phonon modes of Si and Ge. The additional phonon modes at ~12.0 THz match with our simulation results.

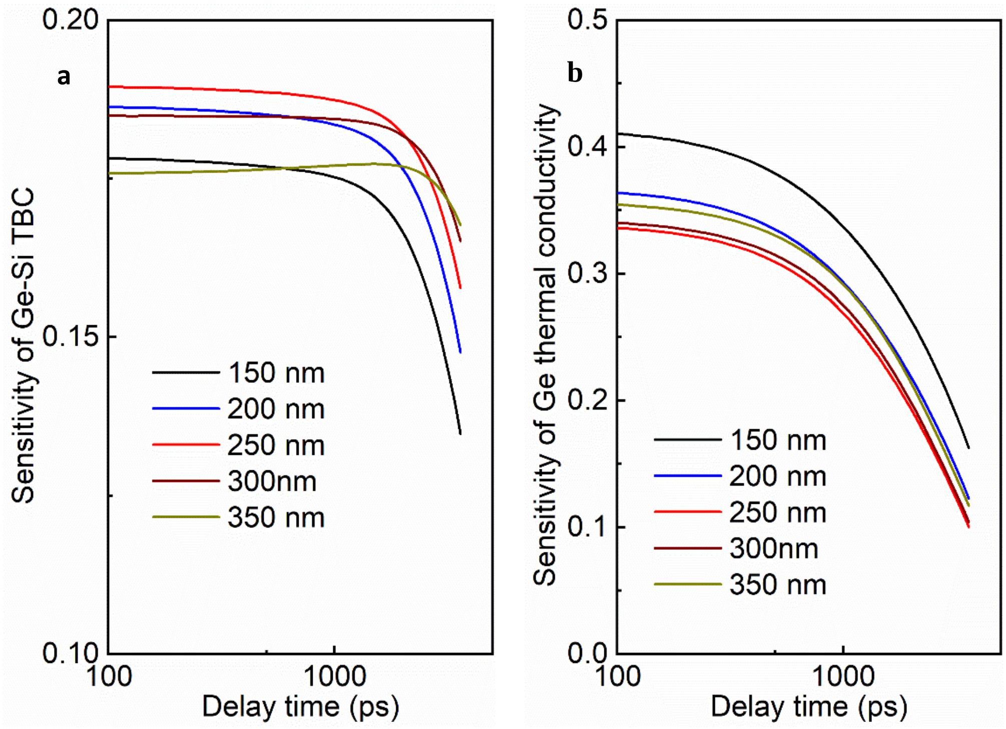

Supplementary Fig.12 TDTR sensitivity as a function of Ge thickness. a Sensitivity of Ge-Si TBC as a function of Ge thickness. **b** Sensitivity of Ge thermal conductivity as a function of Ge thickness.

Supplementary Note 10: Ge thickness optimization in TDTR measurements

By analyzing the TDTR sensitivities of the Si-Ge TBC and the Ge thermal conductivity, we chose 250 nm Ge grown on the Si wafer as the TDTR sample. The TDTR sensitivities of Ge-Si TBC and Ge thermal conductivity as a function of Ge thickness are shown in Supplementary Fig. 12. As we

can see, a Ge thickness of 250 nm gives us the highest sensitivity of Ge-Si TBC and the lowest sensitivity of Ge thermal conductivity.

Supplementary Note 11: laser information

The Raman measurements and TDTR measurements use lasers. The laser information of the Raman system can be found from the product manual (Renishaw InVia). The TDTR laser at Georgia Tech is Spectra-Physics pulsed Mai Tai HP 800-nm laser. Detailed information can be found in the manual of this product. The TDTR laser at UIUC is a Spectra-Physics pulsed Ti-sapphire Tsunami laser. Detailed information can be found in the product manual.

REFERENCES

1 Touloukion, Y. Thermophysical properties of matter. *IFI/PLENUM New York-Washington* **153** (1970).

2 Fulkerson, W., Moore, J., Williams, R., Graves, R. & McElroy, D. Thermal conductivity, electrical resistivity, and seebeck coefficient of silicon from 100 to 1300 K. *Phys. Rev.* **167**, 765 (1968).

3 Holland, M. Analysis of lattice thermal conductivity. *Phys. Rev.* **132**, 2461 (1963).

4 Asheghi, M., Kurabayashi, K., Kasnavi, R. & Goodson, K. Thermal conduction in doped single-crystal silicon films. *J. of Appl. Phys.* **91**, 5079-5088 (2002).

5 Bougher, T. L. *et al.* Thermal boundary resistance in GaN films measured by time domain thermoreflectance with robust Monte Carlo uncertainty estimation. *Nano. and Micro. Thermophys. Eng.* **20**, 22-32 (2016).

6 Kothari, K. & Maldovan, M. Phonon surface scattering and thermal energy distribution in superlattices. *Sci. Rep.* **7**, 1-15 (2017).

7 Cheng, Z. *et al.* Thermal conductance across harmonic-matched epitaxial Al-sapphire heterointerfaces. *Commun. Phys.* **3**, 1-8 (2020).

8 Swartz, E. T. & Pohl, R. O. Thermal boundary resistance. *Rev. of Modern Phys.* **61**, 605 (1989).

9 Monachon, C., Weber, L. & Dames, C. Thermal boundary conductance: A materials science perspective. *Ann. Rev. of Mater. Res.* **46**, 433-463 (2016).

10 Shi, J., Yang, X., Fisher, T. S. & Ruan, X. Dressed and intrinsic thermal boundary conductance across interfaces from a nonequilibrium Landauer approach. *arXiv preprint arXiv:1812.07910* (2018).

11 Tersoff, J. New empirical approach for the structure and energy of covalent systems. *Phys. Rev. B* **37**, 6991 (1988).

12 Stillinger, F. H. & Weber, T. A. Computer simulation of local order in condensed phases of silicon. *Phys. Rev. B* **31**, 5262 (1985).

13 Li, R., Lee, E. & Luo, T. A unified deep neural network potential capable of predicting thermal conductivity of silicon in different phases. *Mater. Today Phys.*, 100181 (2020).

14 Gu, X. & Zhao, C. Thermal conductivity of single-layer MoS2(1−x)Se2x alloys from molecular dynamics simulations with a machine-learning-based interatomic potential. *Comput. Mater. Sci.* **165**, 74-81 (2019).

15 Li, R. *et al.* A deep neural network interatomic potential for studying thermal conductivity of β-Ga2O3. *Appl. Phys. Lett.* **117**, 152102 (2020).

16 Qian, X., Peng, S., Li, X., Wei, Y. & Yang, R. Thermal conductivity modeling using machine learning potentials: application to crystalline and amorphous silicon. *Mater. Today Phys.* **10**, 100140 (2019).

17 Plimpton, S. Fast parallel algorithms for short-range molecular dynamics. *J. of Comput. Phys.* **117**, 1-19 (1995).

18 Kosevich, Y. A. & Strelnikov, I. Extraordinary phonon transmission through hidden lattice-wave nanochannels as resonance quantum phonon tunneling, *AIP Conf. Proceed.*. **2241**, 1, 020023, AIP Publishing LLC, (2020).

19 Chalopin, Y. & Volz, S. A microscopic formulation of the phonon transmission at the nanoscale. *Appl. Phys. Lett.* **103**, 051602 (2013).

20 Gordiz, K. & Henry, A. Phonon transport at crystalline Si/Ge interfaces: the role of interfacial modes of vibration. *Sci. Rep.* **6**, 23139 (2016).

21 Rohskopf, A., Li, R., Luo, T. & Henry, A. A computational method for studying vibrational mode dynamics. *arXiv preprint arXiv:2108.04795* (2021).

22 Glassbrenner, C. J. & Slack, G. A. Thermal conductivity of silicon and germanium from 3 K to the melting point. *Phys. Rev.* **134**, A1058 (1964).

23 Chen, P. *et al.* Role of surface-segregation-driven intermixing on the thermal transport through planar Si/Ge superlattices. *Phys. Rev. Lett.* **111**, 115901 (2013).

24 Chaparro, S. *et al.* Strain-driven alloying in Ge/Si (100) coherent islands. *Phys. Rev. Lett.* **83**, 1199 (1999).

25 Van Nostrand, J. E., Chey, S. J., Hasan, M.-A., Cahill, D. G. & Greene, J. Surface morphology during multilayer epitaxial growth of Ge (001). *Phys. Rev. Lett.* **74**, 1127 (1995).

26 Yan, X. *et al.* Single-defect phonons imaged by electron microscopy. *Nat.* **589**, 65-69 (2021).

27 Wei, S. & Chou, M. Phonon dispersions of silicon and germanium from first-principles calculations. *Phys. Rev. B* **50**, 2221 (1994).

28 Aouissi, M., Hamdi, I., Meskini, N. & Qteish, A. Phonon spectra of diamond, Si, Ge, and α−Sn: Calculations with real-space interatomic force constants. *Phys. Rev. B* **74**, 054302 (2006).
